# Urgent and emergent repair of complex aortic aneurysms using an off-the-shelf branched device

**DOI:** 10.3389/fcvm.2023.1277459

**Published:** 2023-09-22

**Authors:** Petroula Nana, Konstantinos Spanos, Tomasz Jakimowicz, Jose I. Torrealba, Katarzyna Jama, Giuseppe Panuccio, Fiona Rohlffs, Tilo Kölbel

**Affiliations:** ^1^ German Aortic Center, Department of Vascular Medicine, University Heart and Vascular Center UKE Hamburg, Hamburg, Germany; ^2^ Department of General, Vascular and Transplant Surgery, Medical University of Warsaw, Warsaw, Poland

**Keywords:** urgent, ruptured, symptomatic, complex endovascular repair, branched devices, off-theshelf

## Abstract

**Introduction:**

Endovascular repair using off-the-shelf endografts is a viable solution in patients with ruptured or symptomatic complex aortic aneurysms. This analysis aimed to present the peri-operative and follow-up outcomes in urgent and emergent cases managed with the t-Branch multibranched thoracoabdominal endograft.

**Methods:**

Prospectively collected data from all consecutive urgent and emergent cases managed in two aortic centers between January 1st, 2014, to November 30th, 2022, using the t-Branch device (Cook Medical Inc., Bjaeverskov, Denmark) were analyzed. Patients presenting with ruptured aortic complex aneurysms were characterized as emergent and patients with aneurysms >90 mm of diameter, or symptomatic aneurysms were characterized as urgent. Technical success, 30-day mortality, major adverse events (MAE) and spinal cord ischemia (SCI) rates were assessed.

**Results:**

225 patients (36.5% females, 72.5 ± 2.8 years) were included; 73.0% were urgent. The mean aneurysm diameter was 109 ± 3.9 mm and 44.4% were type I–III TAAAs. Females (*p* = .03), para-renal aneurysms (*p* = .02) and ASA score IV (*p* < .001) were more common in emergent cases. Technical success was 97.8%. Thirty-day mortality and MAE rates were 17.8% and 30.6%, respectively. SCI rate was 14.7%, (4.8% paraplegia rate) with 22.2% of patients receiving prophylactic cerebrospinal drainage. Thirty-day mortality (13.3% vs. 26.7%, *p* = .04) and MAE (26.0% vs. 43.0%, *p* = .02) were more common among emergent cases while technical success (97.6% vs. 98.3%, *p* = .9), and SCI (13.3% vs. 18.3%, *p* = .4) were similar. Survival at 12-months was 83.5% (SE 5.9%) for the urgent and 77.1% (SE 8.2%) for the emergent group (log rank, *p* = 0.96).

**Conclusion:**

T-Branch represents an effective and safe solution for the management of urgent and emergent cases with complex aortic aneurysms, with high technical success, promising early mortality and SCI rates.

## Introduction

Mortality in ruptured thoracoabdominal aneurysms (rTAAAs) is high; even when treatment is applied (1, 2). Open surgical repair (OSR) shown higher mortality rates, exceeding 50% in ruptured TAAA with high associated morbidity, especially in terms of renal and cardiac complications (1, 2). In urgent scenarios, endovascular management of TAAAs may provide an early survival benefit, however comparative studies on OSR and complex endovascular aortic repair are still lacking (3–6). Endovascular aortic repair for urgent cases may be achieved by means of different complex techniques, including parallel grafts, surgeon-modified endografts and off-the-shelf devices (7–9). However, limited data exist in the available literature and only few studies focus on urgent complex aortic repair (10, 11).

Promising data, have shown that the off-the-shelf branched devices can be applied in urgent complex aortic cases, including type I–III TAAAs, with low early mortality (10–13). However, there are still issues to be resolved, with spinal cord ischemia (SCI) rates still concerning and raising to 15%, probably as a result of long segment aortic coverage, blood loss, hypotension and the inability to using a staged approach (10, 11). At the same time, some other technical or anatomical issues, as device malrotation, aortoiliac tortuosity, and hostile TV anatomy may affect technical success (10).

The aim of this analysis is to investigate the peri-operative outcomes and follow-up survival in patients managed urgently or emergently for complex aortic aneurysms using the t-Branch device.

## Materials and methods

### Study design and patient cohort

The STrengthening the Reporting of OBservational studies in Epidemiology (STROBE) statement was followed (14). Two aortic-center data, including all urgent and emergent cases, managed between January 1st, 2014, and November 30th, 2022, using the t-Branch device (Cook Medical Inc., Bjaeverskov, Denmark) were prospectively collected in a common database.

All patients managed urgently or emergently for degenerative or post-dissection, thoraco-abdominal aortic (TAAAs), para- and juxta-renal aneurysms, treated with t-Branch, were considered eligible (15). All elective cases as well as patients managed with other off-the-shelf devices, physician modified endografts as well as cases managed with parallel grafts were excluded from this analysis. Patients managed for acute aortic dissections were not included.

All patients underwent clinical evaluation by a vascular specialist. All patients underwent a preoperative computed tomography angiography (CTA) of the entire aorta and iliac arteries with a minimum slice thickness of 3 mm.

Patients with anatomy deemed unsuitable for the t-Branch were managed via open surgery, hybrid repair or other endovascular techniques (16). Hemodynamically unstable patients, that were not considered able to undergo imaging or directly transferred to the hybrid room were excluded from the study (1, 17). All cases were managed in dedicated hybrid operating rooms by the same experienced teams under general anesthesia and with a target activated clotting time of 200–350 s Patients with unsuitable anatomy were managed with open or hybrid repair or other endovascular techniques (16). The technical details of the procedure have been described previously (16).

### Data collection

Patients' sex, age, and comorbidities, including coronary artery disease (CAD), myocardial infarction (MI), previous coronary-aortic bypass (CABG) or coronary stent angioplasty (PTCA), hypertension (HTN), dyslipidemia (DLP), tobacco use (ever or active), chronic obstructive pulmonary disease (COPD), diabetes mellitus (DM), chronic renal disease (CRD), cerebrovascular events [stroke; minor or major, transient ischemic attack (TIA)] and peripheral arterial disease (PAD) were noted and analyzed. Aneurysm type (degenerative or post-dissection), diameter and extension (TAAA, juxta-renal or para-renal aneurysm) were recorded. Crawford's classification was also noted for TAAAs.

Technical success, the duration of operation and fluoroscopy time, volume of contrast, presence of endoleak at completion angiography and its type, as well as the use of cerebrospinal fluid drainage (CSFD) were recorded. Acute kidney injury (AKI), myocardial infarction, stroke (major, minor or TIA), SCI (paraplegia or paraparesis, immediate or delayed), respiratory failure, ischemic colitis, and access complications were collected. Major adverse events, as defined by the reporting standards, were also recorded (19). The length of intensive care unit (ICU) and hospital length of stay (LOS) were both analyzed.

Follow-up included the clinical and imaging re-evaluation, with CTA, within 30 days, at 12th month and yearly, thereafter. Survival was recorded during follow-up. All patients' data were deidentified and inserted in a common database. This study complied with the Declaration of Helsinki and no approval from the local ethics committee was required due to its retrospective nature and unidentifiable information.

### Definitions

All patients managed for ruptured aneurysms were characterized as emergent while cases managed for symptomatic aneurysms without radiologic signs of rupture but with the presence of symptoms, including aneurysm-related pain, peripheral embolization, or diameter over 90 mm were characterized as urgent (15).

Technical success was defined according to the SVS reporting standards as the appropriate endograft deployment with TV patency without evident type I/III endoleak or limb occlusion at final angiography (19). MAEs at 30 days included all-cause mortality, MI, respiratory failure requiring >24 h from anticipated mechanical ventilation or reintubation, renal function decline resulting in >50% reduction in baseline eGFR or new-onset dialysis, bowel ischemia requiring surgical resection or not resolving with medical therapy, major stroke and paraplegia (grade 3) (18). Any new onset, immediate or delayed neurologic deficit of the lower limbs, not attributable to other pathologic entity, including any paraplegia (classes 0–2 of the modified Tarlov's Scoring Scale) or paraparesis (classes 3–5 of the modified Tarlov's Scoring Scale) up to 30 days postoperatively was characterized as SCI (19, 20). AKI was the reduction of the baseline by >25% or any new onset dialysis after repair (19). Myocardial infarction was defined any acute coronary syndrome with typical clinical symptoms and/or electrocardiographic changes and/or troponin elevation.

### Outcomes

Primary endpoints were technical success, 30-day mortality, MAE and SCI rates. Follow-up survival was a secondary endpoint. The cohort was divided into urgent and emergent cases and a comparative analysis was provided.

### Statistical analysis

Continuous data were reported using mean ± standard deviation for normally distributed variables and medians and ranges for non-normally distributed variables. categorical data were expressed as absolute numbers or percentages. Independent two-sample t test was used for normally distributed continuous variables, and the Wilcoxon rank sum test for non-normally distributed continuous and ordinal variables. *P* value was considered significant when it was <.05. Kaplan-Meier estimates were performed to assess survival during follow-up. Estimates were considered reliable in case of standard error <10%. No adjustment for missing was performed. Statistical analysis was performed by SPSS 22.0 for Windows software (IBM Corp, Armonk, NY).

## Results

### Patients' characteristics

Since 2014, 1,658 patients were managed in both departments using fenestrated and branched endovascular repair; among them 631 were managed with t-Branch in elective and urgent/emergent setting. In total, 225 urgent and emergent patients were managed using the t-branch device; 165 (73.3%) underwent urgent repair. For the total cohort, the mean age was 72.5 ± 2.8 years and females represented 36.5%. ASA score III was recorded in 64.0% of the cases while 20.4% were ASA score IV. Among comorbidities, HTN was the most common (87.6%), followed by smoking in 52.4% of patients; among them 45.8% were active smokers. Among patients, 12.4% had a previous history of endovascular abdominal aortic repair (EVAR) while 12.4% had undergone previous thoracic repair. The mean aneurysm diameter was 109 ± 3.9 mm and 100 (44.4%) patients were managed for type I–III TAAAs. The remaining baseline characteristics of the total cohort are presented in Table 1.

**Table 1:** The distribution of the pre-operative characteristics of the total cohort including urgent and emergent cases.

Baseline characteristics	Total cohort (225 patients)
Age (years)	72.5 ± 2.8
Females	82 (36.5%)
Setting	
Urgent	165 (73.3%)
Emergent	60 (26.7%)
Aneurysm characteristics	
Aneurysm diameter (mm)	109.0 ± 3.9
Thoracoabdominal aneurysm	196 (87.1%)
I	12 (5.3%)
II	38 (16.9%)
III	50 (22.2%)
IV	85 (37.8%)
V	11 (4.9%)
I-III	100 (57.1%)
Pararenal aneurysm	17 (7.6%)
Juxtarenal aneurysm	12 (5.3%)
Comorbidities	
CAD	106 (47.1%)
Previous MI	40 (17.8%)
CABG	23 (10.2%)
PTCA	31 (13.8%)
HTN	197 (87.6%)
CRD	81 (36.0%)
Dialysis dependent	5 (2.2%)
COPD	43 (19.1%)
DLP	112 (49.8%)
Smoking	118 (52.4%)
Active smoking	54 (24.0%)
Stroke	29 (12.9%)
TIA	4 (1.8%)
PAD	57 (25.3%)

CABG, coronary-aortic bypass graft; CAD, coronary artery disease; COPD, chronic obstructive pulmonary disease; CRD, chronic renal disease; DLP, dyslipidemia; HTN, hypertension; MI, myocardial infarction; PAD, peripheral arterial disease; PTCA, percutaneous transcatheter coronary angioplasty; TIA, transient ischemic attack.

Technical success was 93.7% while eleven technical failures were reported: six due type III endoleaks, three due to endoleak type I, three to unsuccessful TV catheterizations (one due to celiac artery and two due to renal artery failed catheterizations), one due to unintentional CT occlusion and one due to TV iatrogenic trauma. In total, 833 TV were successfully revascularized. The infrarenal aorta was selected as the preferential landing zone in 24 patients (10.7%) while in the remaining cases, a bifurcated distal extension was used. In total, 50 patients received a prophylactic CSFD (22.2%); in no case a therapeutic CSFD was used. Eight patients underwent debranching (3.6%). The estimated operational and fluoroscopy times were 265 ± 47 min and 38.3 ± 10.8 min, respectively while the mean contrast volume was 228 ± 27 ml. For endoleaks detected at completion angiography, no further intervention was performed, except four cases of type III endoleaks that were managed with additional balloon angioplasty. In the remaining cases, a “wait and watch” approach was used until the predischarge CTA. All type Ia endoleaks were eliminated by that time.

Thirty-day mortality was 17.8%. MAE rate was 30.6% while SCI rate was 14.7%. In total, 33 patients developed SCI: 4.8% with paraplegia while the remaining patients presented paraparesis. Among SCI patients, 67% presented symptoms immediately after the procedure and the remaining had delayed SCI. AKI was recorded in 17.8% of cases; with 2% of patients needing permanent dialysis. Two patients (0.9%) presented signs of post-operative MI. A detailed analysis of the total cohort outcomes is presented in Table 2. Thirty-day reinterventions were needed in 30 cases (18%), among them 60% was TV-related. In total, 65 (28.9%) patients presented access complications and 38 (16.9%) needed a secondary access intervention.

**Table 2:** The distribution of post-operative early outcomes, including technical success and mortality of the total cohort.

Early outcomes	Total cohort (225 cases)
Technical success	211 (93.7%)
TV failed revascularization	3 (1.3%)
Endoleak at completion angiography	
Endoleak type I	3 (1.3%)
Endoleak type II	32 (14.2%)
Endoleak type III	8 (3.6%)
Mortality	38 (16.9%)
In-hospital	40 (17.8%)
MAE	69 (30.7%)
SCI	33 (14.7%)
Paraplegia	11 (4.8%)
Delayed	11 (4.8%)
AKI	39 (17.3%)
Dialysis	10 (4.4%)
MI	2 (0.9%)
Respiratory failure	10 (4.4%)
Stroke	10 (4.4%)
Major	9 (4.0%)
Bowel ischemia	6 (2.7%)
Needing resection	1 (0.4%)
Access complications	38 (16.9%)
Infection needing surgery	1 (0.4%)
Hematoma/pseudoaneurysm needing surgery	37 (16.5%)

AKI, acute kidney injury; MAE, major adverse events; MI, myocardial infarction; SCI, spinal cord ischemia; TV, target vessel.

### Comparative early outcomes between urgent and emergent cases

The mean age among urgent cases was 72.3 ± 3.3 years vs. 73.0 ± 5.6 years in the emergent cohort (*p* = .8). Female sex was more common among emergent cases (41.2% vs. 25.0%, *p* = .03). Mean aneurysm diameter was 102 ± 42 mm and 127 ± 85 mm (*p* = .09) for the urgent and emergent group, respectively. Aneurysm type distribution did not differentiate between the groups for type I–III TAAA; 45.5% in urgent vs. 41.7% in emergent (*p* = .8) but did significantly differ in pararenal aneurysms with 4.8% in urgent vs. 15% in emergent cases (*p* = .02).

Regarding comorbidities, cohorts presented similar findings, except the distribution of hypertension and chronic renal disease, which were higher in the urgent group (90.3% vs. 80.0%, *p* = .04 and 38.2% vs. 30%, *p* = .04, respectively). Previous thoracic and endovascular aortic aneurysm repair were similar between groups (*p* = .2 and *p* = .8, respectively). ASA score IV was more common among patients managed emergently (14.5% vs. 36.7%, *p* < .001). The distribution of the comparative pre-operative details is presented in Table 3.

**Table 3:** The distribution of pre-operative characteristics between urgent and emergent cases.

Baseline characteristics	Urgent cohort (165 patients)	Emergent cohort (60 cases)	*P*
Age (years)	72.3 ± 3.3	73.0 ± 5.6	.8
Females	67 (41.2%)	15 (25.0%)	.03
Aneurysm characteristics			
Aneurysm diameter (mm)	102 ± 42	127 ± 85	.09
TAAA	150 (90.9%)	46 (76.7%)	.5
I	9 (5.5%)	3 (5.0%)	.9
II	25 (15.2%)	13 (21.7%)	.3
III	41 (24.8%)	9 (15.0%)	.09
IV	69 (41.8%)	16 (26.7%)	.03
V	6 (3.6%)	5 (8.3%)	.2
I-III	75 (45.5%)	25 (41.7%)	.8
Pararenal aneurysm	8 (4.8%)	9 (15.0%)	.02
Juxtarenal aneurysm	7 (4.2%)	5 (8.3%)	.3
ASA score			
II	22 (13.3%)	7 (11.7%)	.8
III	114 (69.0%)	30 (50.0%)	.2
IV	24 (14.5%)	22 (36.7%)	<.001
Comorbidities			
CAD	80 (48.4%)	26 (43.3%)	.5
Previous MI	30 (18.2%)	10 (16.7%)	.8
CABG	17 (10.3%)	6 (10.0%)	.9
PTCA	27 (16.4%)	4 (6.7%)	.9
HTN	149 (90.3%)	48 (80.0%)	.04
CRD	63 (38.2%)	18 (30.0%)	.04
Dialysis dependent	4 (2.4%)	1 (1.7%)	.7
COPD	33 (20.0%)	10 (16.7%)	.6
DLP	89 (53.9%)	23 (38.3%)	.2
Smoking	88 (53.3%)	30 (50.0%)	.8
Active smoking	37 (22.4%)	17 (28.3%)	.4
Diabetes	24 (14.5%)	5 (8.3%)	.2
Stroke	24 (14.5%)	5 (8.3%)	.2
TIA	3 (1.8%)	1 (1.7%)	.9
PAD	77 (46.7%)	30 (50.0%)	.7

CABG, coronary-aortic bypass graft; CAD, coronary artery disease; COPD, chronic obstructive pulmonary disease; CRD, chronic renal disease; DLP, dyslipidemia; HTN, hypertension; MI, myocardial infarction; PAD, peripheral arterial disease; PTCA, percutaneous transcatheter coronary angioplasty; TAAA, thoraco-abdominal aortic aneurysm; TIA, transient ischemic attack.

Five patients underwent a left carotid subclavian bypass in the urgent group (3.0%) and three in the emergent group (5.0%, *p* = .5) while prophylactic CSFD was used equally in both groups (23.0% vs. 20.0%, *p* = .7). Technical success was similar; 93.3% among urgent and 95.0% among emergent cases (*p* = .9). Procedural times were similar between groups (264 ± 54 min vs. 267 ± 92 min, *p* = .8) but fluoroscopy and contrast use were higher in the urgent group (38.7 ± 13.2 min vs. 37.1 ± 17.6 min, *p* = .04 and 39 ± 13 ml vs. 37 ± 18 ml, *p* = <.001). Landing to the distal abdominal aorta (above the aortic bifurcation) was performed in 9.0% of urgent vs. 15.0% of emergent cases (*p* = .3). The incidence of type I, II and III endoleak at completion angiography was similar between groups (*p* = .9).

Thirty-day mortality was 13.3% in urgent and 26.7% in emergent patients (*p* = .04). In-hospital mortality was also higher in the emergent cohort (*p* = .04). The MAE rate was also higher in emergent cases; 26.0% vs. 43.0% (*p* = .02). There were no significant differences in SCI among groups, with 13.3% in the urgent vs. 18.3% in the emergent group (*p* = .4). No difference was detected in paraplegia (*p* = 1.0), AKI rates (*p* = .3), and need for dialysis rates (*p* = .1). The only difference was recorded in bowel ischemia; *p* = .03.

Thirty-day reinterventions were needed in 13.9% in the urgent and 11.7% in the emergent group (*p* = .7). TV-related reintervention rates were 7.8% vs. 8.3% (*p* = .9). There were no significant differences between the median (IQR) ICU stay (7 (9) days in urgent vs. 9 (7) days in emergent, *p* = .2) and LOS (9 I(10) days in urgent vs. 10 (12) days in emergent cases, *p* = .7). All remaining adverse events are presented in Table 4.

**Table 4:** The distribution of post-operative early outcomes, including technical success and mortality between urgent and emergent cases.

Early outcomes	Urgent cohort (165 cases)	Emergent cohort (60 cases)	*P*
Technical success	154 (93.3%)	57 (95.0%)	.7
TV failed revascularization	2 (1.2%)	1 (1.8%)	.8
Endoleak at completion angiography			
Endoleak type I	2 (1.2%)	1 (1.8%)	.8
Endoleak type II	23 (13.9%)	9 (15.0%)	.9
Endoleak type III	6 (3.6%)	2 (3.3%)	.9
Mortality	22 (13.3%)	16 (26.7%)	.04
In-hospital	24 (14.5%)	16 (26.7%)	.04
MAE	43 (26.0%)	26 (43.0%)	.02
SCI	22 (13.3%)	11 (18.3%)	.4
Paraplegia	8 (4.8%)	3 (5.0%)	1.0
Delayed	9 (5.5%)	2 (3.3%)	.5
AKI	26 (15.8%)	13 (21.7%)	.3
Dialysis	5 (3.0%)	5 (8.3%)	.1
MI	2 (1.2%)	0 (0.0%)	.3
Respiratory failure	9 (5.5%)	1 (1.7%)	.2
Stroke	8 (4.8%)	2 (3.3%)	.6
Major	7 (4.2%)	2 (3.3%)	.8
Bowel ischemia	2 (1.2%)	4 (6.7%)	.03
Needing resection	0 (0.0%)04	1 (1.7%)	.5

AKI, acute kidney injury; MAE, major adverse events; MI, myocardial infarction; SCI, spinal cord ischemia; TV, target vessel.

### Follow-up survival for the total cohort and subgroups

**Figure 1:**
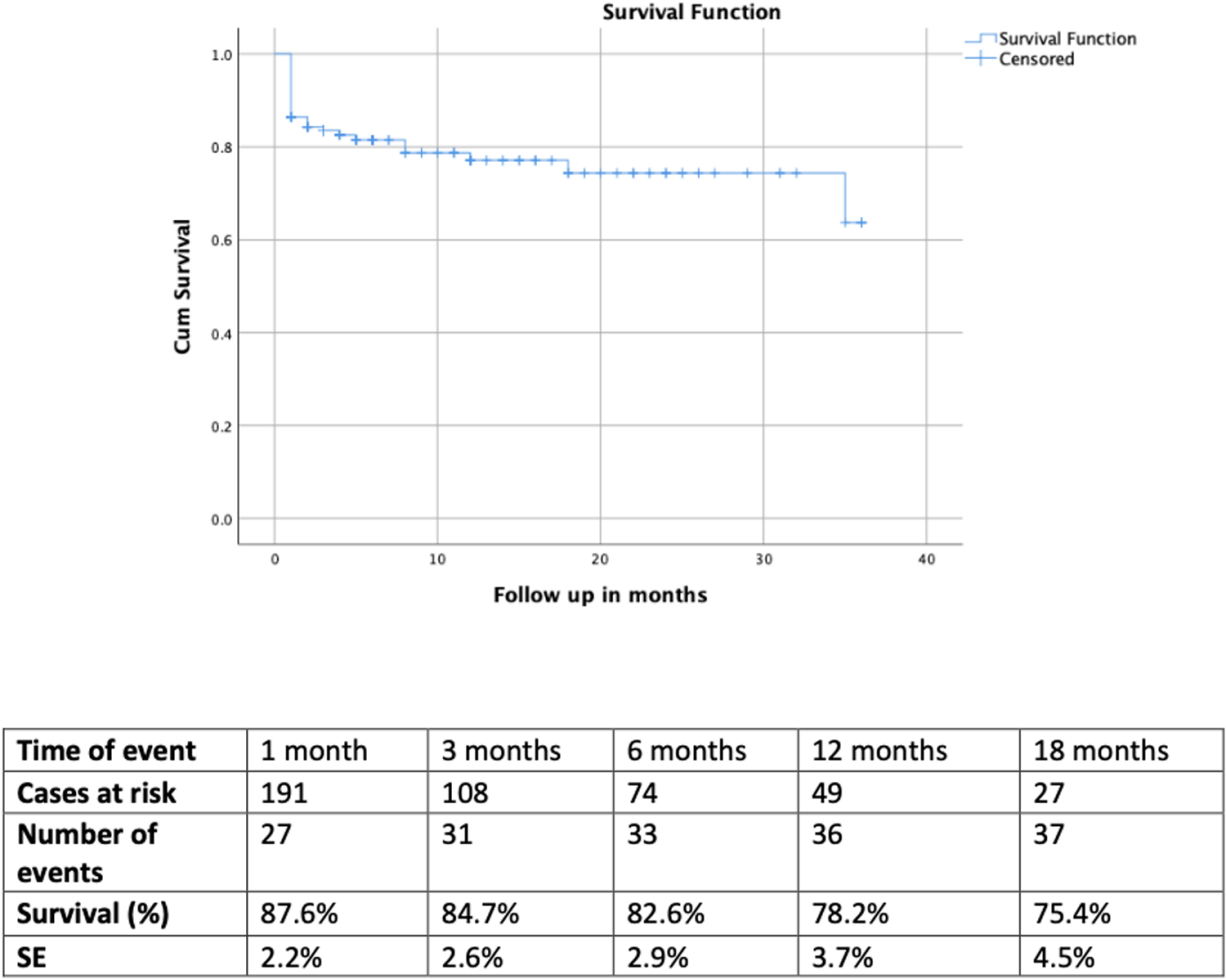
The kaplan meier curve depicts the survival of the total cohort. The estimated rate was rates was 75.4% (SE 4.5%) at 18 months. No death was aorta related during the available follow-up.

**Figure 2:**
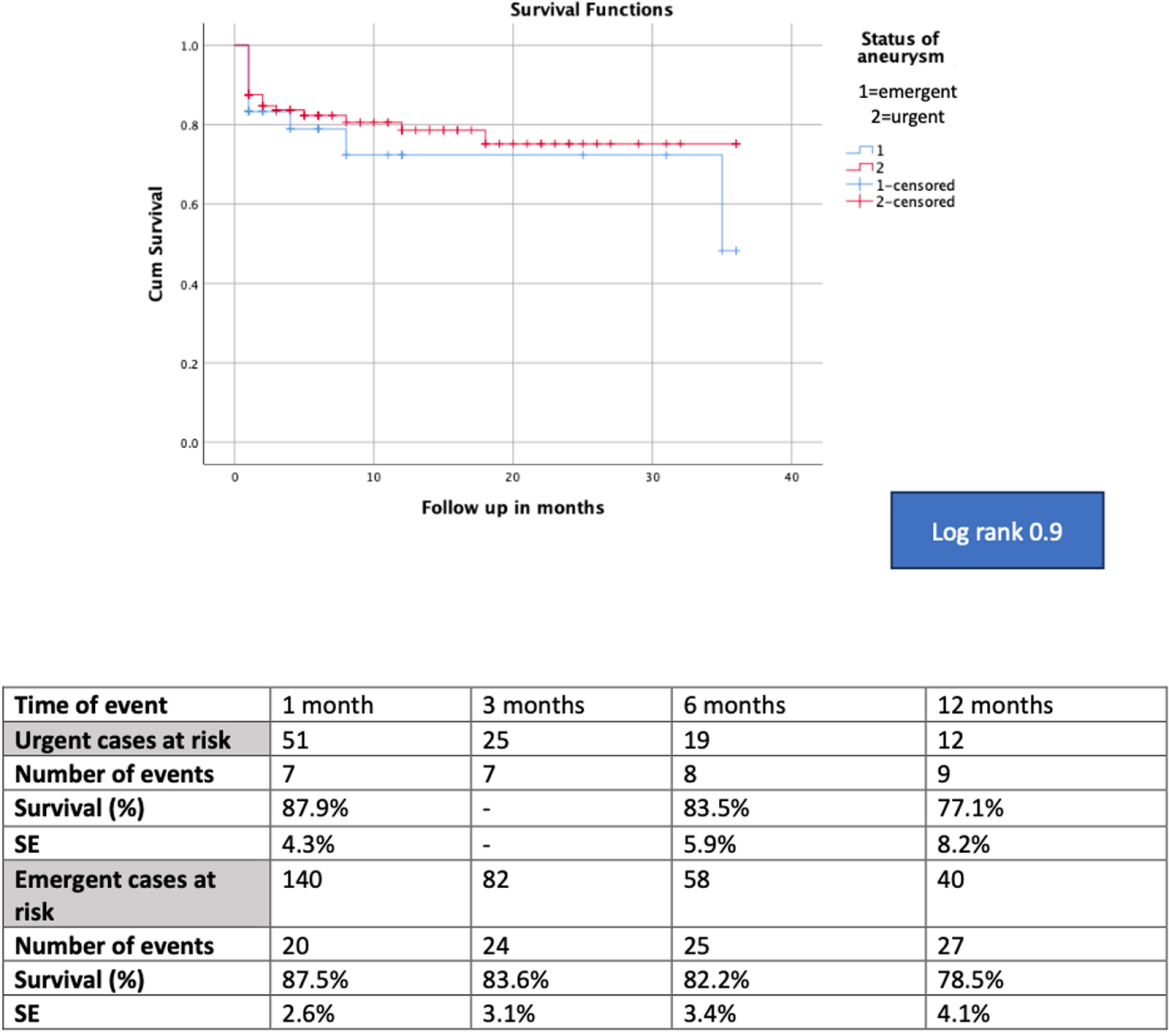
The kaplan meier curve of the comparative survival showed no difference between groups (log rank, *p* = .9).

The mean follow up was 7.2 ± 4.0 months for the total cohort and 7.8 ± 5.0 months and 5.4 ± 5.5 months for the urgent and emergent group respectively (*p* = .09). For the total cohort, the survival rates were 82.6% (SE 2.9%) at 6 months, 78.2% (SE 3.7%) at 12 months and 75.4% (SE 4.5%) at 18 months. The estimated survival for the urgent and emergent cohort was 83.5% (SE 5.9%) and 78.9% (SE 4.1%) at 12 months, respectively. When comparing both groups, no difference was detected in survival (log rank, *p* = .9). The Kaplan Meier curves of the total cohort and comparative survival are presented in Figures 1, 2. No death was aorta related during the available follow-up.

## Discussion

Complex endovascular aortic repair of juxtarenal aortic aneurysms has gained popularity during the last decade while a decision between open and endovascular repair in ruptured cases should be based on patients' anatomy, general status and preferences (21). Regarding TAAAs, specific recommendations on complex endovascular aortic repair under urgent circumstances are lacking (22). In this analysis, the t-Branch device was used to treat a variety of emergent and urgent cases, including thoracoabdominal, juxta- and para-renal aneurysms with an early mortality of 16.8% for the total cohort and 26% for the emergent cases. These findings align to the available literature reporting on 17% 30-day and 25% in-hospital mortality in patients needing urgent complex endovascular repair (23, 24).

Off-the-shelf solutions represent the main approach in patients needing urgent complex aortic repair, including parallel grafts, physician modified endografts with in-situ fenestrations or on-table modifications, and off-the shelf branched devices (21, 22). Off-the-shelf branched endografts may be utilized with good similar technical success in urgent and elective cases, when anatomically feasible (25–28). However, according to the available literature, only a percentage of cases will be suitable for these devices and the feasibility range fluctuates widely between 40% and 90% (29–31). When reported on female patients, the feasibility is even lower, reaching only 22% (32). The application of off-the-shelf endografts in females has been related to 16% mortality in mixed cohorts and 22% in exclusively urgent cases (33). Additional modifications in the available branched devices or newer generations may be able to expand the applicability of the technique in a broader patient population.

Endovascular management of ruptured AAA have demonstrated improved outcomes during last decade, showing that early mortality in ruptured AAA cases is estimated at 20% (34–36). Compared to standard endovascular repair for ruptured AAA and taking into consideration that 45% of patients in the current study were managed for extensive type I–III TAAAs, the early mortality could be considered acceptable both for urgent and emergent repair. Similar findings confirm the safety of the t-Branch device in the short-term follow-up with mortality rates ranging between 6% and 25% in cohorts mainly represented by urgent TAAAs (10, 16, 18). Early mortality was significantly higher within the emergent cohort in the current study, suggesting that some factors related to the ruptured aortic status, including hemodynamic instability, might affect early findings, while urgent, non-ruptured cases, represent rather an intermediate condition with better outcomes than emergent cases. However, due to the need for faster decision making and management as well, the associated perioperative mortality might be higher than in elective cases (37).

MAE affected one of three cases in the total cohort and are within the reported range in the literature (10). Among these events, AKI was recorded in 17.8% of patients while permanent dialysis was needed only in 2%; aligned to the previously published data (10). For SCI, the reported adverse event rate is ranging between 5% and 17% in the current literature while a statistically significant difference has been reported between patients managed for TAAAs under urgent and emergent setting (10, 11, 18). The emergent setting appears to affect outcomes, with a nearly fourfold increased likelihood of SCI among these patients (18). Factors related to hemodynamic stability, pre-operative anemia, lack of staged repair and the lower use of CSFD potentially affected these findings and need further investigation. Systematically, preventive conservative measures were applied and probably achieved to decrease the difference of SCI between urgent and emergent cases (38).

Reinterventions during the 30-day follow-up are not uncommon after t-Branch for urgent repair (10, 11, 18). The reported rate is up to 25% while freedom from re-intervention during the 24-month follow-up has been estimated between 25% and 40% (11, 18). In the current cohort, the 30-day re-intervention rate was 18%, and 60% of them were TV related. Emergent repair and technical failure seem to be related in t-Branch cohorts with technical failure significantly relating to TV occlusion (10). TV related failures were the most common reason for technical failure in this analysis and TV-related reinterventions were the leading cause of early reintervention. The reintervention rates of both groups remained acceptable, probably affected by the previous experience of the participating departments with the t-Branch device in elective cases and appropriate patient selection (39, 40).

Endovascular repair provides beneficial survival compared to OSR in complex aortic cases; especially when focusing on more recent studies (15). On complex aortic cases and mixed cohorts including elective and urgent cases, endovascular management has achieved similar early and mid-term survival benefit to OSR whereas after 2 years of follow-up, OSR seems to have lower mortality rates (5, 41, 42). Specific comparative data focusing on urgent complex aortic aneurysms are lacking, however according to current experience, branched endovascular repair for ruptured TAAA has been related to a survival rate ranging between 60% and 89% during the midterm follow-up (10, 11, 18, 42). In this analysis, no difference was detected between emergent und urgent cases at 12 months of follow-up. Further long-term data is needed to firmly support these findings.

## Limitations

The retrospective design and sample size should be taken under consideration when evaluating the findings of this analysis. Potential type II errors cannot be excluded. The absence of anatomical details, including iliac access (atheromatosis, tortuosity) as well as aortic anatomy should be acknowledged while the application of the device within the IFUs was not examined. TAAAs, juxta- and para-renal aneurysms were included in this analysis and potentially affected our findings. Data on patients managed with open or hybrid repair were not included in the current database and were not available for further assessment. However, all patients were managed with the same device, technique and by the same experienced teams. The selective application of cerebrospinal fluid drainage, as well as the different protocols for its use between the centers may also inserted bias, especially on SCI findings. Long-term follow-up data on survival are missing and firm conclusions cannot be extracted.

## Conclusions

The t-Branch device represents an effective and safe solution for the management of urgent and emergent patients needing complex endovascular aortic aneurysm repair with high technical success, less than 20% early mortality and acceptable SCI rates, considering the nature of the intervention. Emergent cases presented higher mortality and MAE rates.

## Data Availability

The original contributions presented in the study are included in the article/Supplementary Materials, further inquiries can be directed to the corresponding author.

## References

[1] Jr JA C Dimick JB Wainess RM Henke PK Stanley JC Upchurch Jr GR. Ruptured thoracoabdominal aortic aneurysm treatment in the United States: 1988 to 1998. J Vasc Surg. (2003) 38:319–22. 10.1016/s0741-5214(03)00227-1 12891114

[2] Moulakakis KG Karaolanis G Antonopoulos CN Kakisis J Klonaris C Preventza O Open repair of thoracoabdominal aortic aneurysms in experienced centers. J Vasc Surg. (2018) 68:634–45. 10.1016/j.jvs.2018.03.410 30037680

[3] Sultan S Concannon J Veerasingam D Tawfick W McHugh P Jordan F Endovascular versus conventional open surgical repair for thoracoabdominal aortic aneurysms. Cochrane Database Syst Rev. (2022) 4:CD012926. 10.1002/14651858.CD012926.pub2 35363887 PMC9370075

[4] Fionnula J FitzGibbon B Kavanagh EP McHugh P Veersingam D Sultan S Endovascular versus open surgical repair for complicated chronic type B aortic dissection. Cochrane Database Syst Rev. (2021) 12:CD012992. 10.1002/14651858.CD012992.pub2 34905228 PMC8670553

[5] Latz CA Boitano LT Tanious A Wang LJ Schwartz SL Pendleton AA Endovascular versus open repair for ruptured Complex abdominal aortic aneurysms: a propensity weighted analysis. Ann Vasc Surg. (2020) 68:34–43. 10.1016/j.avsg.2020.04.073 32439527

[6] Soden PA Zettervall SL Ultee KH Darling JD Buck DB Hile CN Outcomes for symptomatic abdominal aortic aneurysms in the American college of surgeons national surgical quality improvement program. J Vasc Surg. (2016) 64:297–305. 10.1016/j.jvs.2016.02.055 27146791 PMC5065370

[7] Spanos K Kölbel T Heidemann F Debus ES Rohlffs F Tsilimparis N. Early and mid-term durability of surgeon-modified and custom-made fenestrated devices for the treatment of complex aortic pathology. Ann Vasc Surg. (2022) 83:212–21. 10.1016/j.avsg.2021.12.006 34954035

[8] Le Houérou T Fabre D Alonso CG Brenot P Bourkaib R Angel C In situ antegrade Laser fenestrations during endovascular aortic repair. Eur J Vasc Endovasc Surg. (2018) 56:356–62. 10.1016/j.ejvs.2018.05.014 30196815

[9] Bannazadeh M Beckerman WE Korayem AH McKinsey JF. Two-year evaluation of fenestrated and parallel branch endografts for the treatment of juxtarenal, suprarenal, and thoracoabdominal aneurysms at a single institution. J Vasc Surg. (2020) 71:15–22. 10.1016/j.jvs.2019.03.058 31718954

[10] Gallitto E Gargiulo M Freyrie A Ancetti S Faggioli G Stella A Off-the-shelf multibranched endograft for urgent endovascular repair of thoracoabdominal aortic aneurysms. J Vasc Surg. (2017) 66:696–704. 10.1016/j.jvs.2016.12.129 28366305

[11] Gallitto E Faggioli G Spath P Pini R Mascoli C Logiacco A Urgent endovascular repair of thoracoabdominal aneurysms using an off-the-shelf multibranched endograft. Eur J Cardiothorac Surg. (2022) 61:1087–96. 10.1093/ejcts/ezab553 34964451

[12] Piazza M Squizzato F Pratesi G Tshomba Y Gaggiano A Gatta E Off the shelf preloaded inner branch endograft for the treatment of complex aortic pathologies in the Italian branched registry of E-nside EnDograft (INBREED). Eur J Vasc Endovasc Surg. (2023) 65:811–7. 10.1016/j.ejvs.2023.02.076 36871927

[13] Biggs JH Tenorio ER DeMartino ER DeMartino RR Oderich GS Mendes BC. Outcomes following urgent fenestrated-branched endovascular repair for pararenal and thoracoabdominal aortic aneurysms. Ann Vasc Surg. (2022) 85:87–95. 10.1016/j.avsg.2022.05.003 35595206

[14] von Elm E Altman DG Egger M Pocock SJ Gøtzsche PC Vandenbroucke JP The strengthening the reporting of observational studies in epidemiology (STROBE) statement: guidelines for reporting observational studies. Int J Surg. (2014) 12(12):1495–9. 10.1016/j.ijsu.2014.07.013 25046131

[15] Kölbel T Spanos K Jama K Behrendt CA Panuccio G Eleshra A Early outcomes of the t-branch off-the-shelf multi-branched stent graft in 542 patients for elective and urgent aortic pathologies: a retrospective observational study. J Vasc Surg. (2021) 74:1817–24. 10.1016/j.jvs.2021.05.041 34171424

[16] Spanos K Kölbel T Theodorakopoulou M Heidemann F Rohlffs F Debus ES Early outcomes of the t-branch off-the-shelf multibranched stent-graft in urgent thoracoabdominal aortic aneurysm repair. J Endovasc Ther. (2018) 25:31–9. 10.1177/1526602817747282 29235388

[17] Harris DG Garrido D Oates CP Kalsi R Huffner ME Toursavadkohi S Repair of ruptured abdominal aortic aneurysm after cardiac arrest. J Vasc Surg. (2016) 64:1497–502. 10.1016/j.jvs.2016.05.085 27473775

[18] Acher C Acher CW Castello Ramirez MC Wynn M. Operative mortality and morbidity in ruptured abdominal aortic aneurysms in the endovascular age. Ann Vasc Surg. (2020) 66:70–6. 10.1016/j.avsg.2019.10.073 31676380

[19] Oderich GS Forbes TL Chaer R Davies MG Lindsay TF Mastracci T Reporting standards for endovascular aortic repair of aneurysms involving the renal-mesenteric arteries. J Vasc Surg. (2021) 73:4S–52S. 10.1016/j.jvs.2020.06.011 32615285

[20] Lombardi JV Hughes GC Appoo JJ Bavaria JE Beck AW Cambria RP Society for vascular surgery (SVS) and society of thoracic surgeons (STS) reporting standards for type B aortic dissections. J Vasc Surg. (2020) 71:723–47. 10.1016/j.jvs.2019.11.013 32001058

[21] Wanhainen A Verzini F Van Herzeele I Allaire E Bown M Cohnert T Editor’s choice—European society for vascular surgery (ESVS) 2019 clinical practice guidelines on the management of abdominal aorto-iliac artery aneurysms. Eur J Vasc Endovasc Surg. (2019) 57:8–93. 10.1016/j.ejvs.2018.09.020 30528142

[22] Riambau V Böckler D Brunkwall J Cao P Chiesa R Coppi G Editor’s choice—management of descending thoracic aorta diseases: clinical practice guidelines of the European society for vascular surgery (ESVS). Eur J Vasc Endovasc Surg. (2017) 53:4–52. 10.1016/j.ejvs.2016.06.005 28081802

[23] Gallitto E Faggioli G Pini R Mascoli C Freyrie A Vento V Total endovascular repair of contained ruptured thoracoabdominal aortic aneurysms. Ann Vasc Surg. (2019) 58:211–21. 10.1016/j.avsg.2018.12.065 30763709

[24] Ferrer C Orrico M Spararo C Coscarella C Ronchey S Marino M Outcomes of multibranched off-the-shelf stent graft in elective and urgent/emergent repair of complex aortic aneurysms with narrow internal aortic lumen. J Vasc Surg. (2022) 76:326–34. 10.1016/j.jvs.2022.03.007 35314297

[25] Silingardi R Gennai S Leone N Gargiulo M Faggioli G Cao P Standard “off-the-shelf” multibranched thoracoabdominal endograft in urgent and elective patients with single and staged procedures in a multicenter experience. J Vasc Surg. (2018) 67:1005–16. 10.1016/j.jvs.2017.08.068 29097044

[26] Tsilimparis N Heidemann F Rohlffs F Diener H Wipper S Debus ES Outcome of surgeon-modified fenestrated/branched stent-grafts for symptomatic complex aortic pathologies or contained rupture. J Endovasc Ther. (2017) 24:825–32. 10.1177/1526602817729673 28874089

[27] Georgiadis GS van Herwaarden JA Antoniou GA Hazenberg CEVB Giannoukas AG Lazaridis MK Systematic review of off-the-shelf or physician-modified fenestrated and branched endografts. J Endovasc Ther. (2016) 23:98–109. 10.1177/1526602815611887 26496957

[28] Bilman V Rinaldi E Loschi D Scheick-Yousif B Melissano G. Suitability of current off-the-shelf devices for endovascular TAAA repair: a systematic review. J Cardiovasc Surg (Torino). (2023). 10.23736/S0021-9509.23.12704-2 37199677

[29] Bilamn V Cambiaghi T Grandi A Carta N Melissano G Chiesa R Anatomical feasibility of a new off-the-shelf inner branch stent graft (E-nside) for endovascular treatment of thoraco-abdominal aneurysms. J Vasc Surg. (2020) 58:1296–303. 10.1093/ejcts/ezaa276 33057585

[30] Bertoglio L Grandi A Carta N Cambiaghi T Bilman V Melissano G Comparison of anatomic feasibility of three different multibranched off-the-shelf stent-grafts designed for thoracoabdominal aortic aneurysms. J Vasc Surg. (2021) 74:1472–82. 10.1016/j.jvs.2021.04.066 34023432

[31] Cambiaghi T Grandi A Bilman A Melissano A Chiesa R Bertoglio L. Anatomic feasibility of the investigational GORE EXCLUDER thoracoabdominal branch endoprosthesis (TAMBE), off-the-shelf multibranched endograft for the treatment of pararenal and thoracoabdominal aortic aneurysms. J Vasc Surg. (2021) 73:22–30. 10.1016/j.jvs.2020.03.056 32360681

[32] Grandi A Carta N Cambiaghi T Bilman V Melissano G Chiesa R Sex-Related anatomical feasibility differences in endovascular repair of thoracoabdominal aortic aneurysms with a multibranched stent-graft. J Endovasc Ther. (2021) 28:283–94. 10.1177/1526602820964916 33045878

[33] Nana P Spanos K Kölbel T Panuccio G Jama K Jakimowicz T Early and mid-term outcomes of females treated with t-branch off the shelf device. Ann Vasc Surg. (2023) 95:32–41. 10.1016/j.avsg.2023.05.033 37268105

[34] Alsusa H Shahid A Antoniou GA. A comparison of endovascular versus open repair for ruptured abdominal aortic aneurysm—meta-analysis of propensity score-matched data. Vascular. (2022) 30:628–38. 10.1177/17085381211025168 34126813

[35] Spanos K Nana P Behrendt CA Kouvelos G Panuccio G Heidemann F Management of abdominal aortic aneurysm disease: similarities and differences among cardiovascular guidelines and NICE guidance. J Endovasc Ther. (2020) 27:889–901. 10.1177/1526602820951265 32813590

[36] Wang LJ Locham S Al-Nouri O Eagleton MJ Clouse WD Malas MB. Endovascular repair of ruptured abdominal aortic aneurysm is superior to open repair: propensity-matched analysis in the vascular quality initiative. J Vasc Surg. (2020) 72:498–507. 10.1016/j.jvs.2019.11.063 32273221

[37] Eleshra A Hatm M Spanos K Panuccio G Rohlffs F Debus ES Early outcomes of t-branch off-the-shelf multibranched stent graft in urgent and emergent repair of thoracoabdominal aortic aneurysms. J Vasc Surg. (2022) 75:416–24. 10.1016/j.jvs.2021.07.237 34480993

[38] Luebke T Brunkwall J. Risk-Adjusted meta-analysis of 30-day mortality of endovascular versus open repair for ruptured abdominal aortic aneurysms. Ann Vasc Surg. (2015) 29:845–63. 10.1016/j.avsg.2014.12.014 25725271

[39] Locatelli F Nana P Le Houérou T Guirimand A Nader M Gaudin A Spinal cord ischemia rates and prophylactic spinal drainage in patients treated with fenestrated and branched endovascular repair for thoracoabdominal aneurysms. J Vasc Surg. (2023) So741-5214(23)01287. 10.1016/j.jvs.2023.06.022 37315908

[40] Alberga AJ von Meijenfeldt GCI Rastogi V de Bruin JL Wever JJ van Herwaarden JA Association of hospital volume with perioperative mortality of endovascular repair of Complex aortic aneurysms: a nationwide cohort study. Ann Surg. (2021). 10.1097/SLA.0000000000005337 34913891

[41] Kontopodis N Galanakis N Ioannou CV Tsetis D Becquemin JP Antoniou GA. Time-to-event data meta-analysis of late outcomes of endovascular versus open repair for ruptured abdominal aortic aneurysms. J Vasc Surg. (2021) 74:628–38. 10.1016/j.jvs.2021.03.019 33819523

[42] Zhang K Zheng H Hu Z Liang Z Hao Y Chen Z. Endovascular repair versus open surgical repair for Complex abdominal aortic aneurysms: a systematic review and meta-analysis. Ann Vasc Surg. (2023) 93:355–68. 10.1016/j.avsg.2022.06.102 35926793

[43] Hongku K Sonesson B Björses K Holst J Resch T Dias NV. Mid-term outcomes of endovascular repair of ruptured thoraco-abdominal aortic aneurysms with off the shelf branched stent grafts. Eur J Vasc Endovasc Surg. (2018) 55:377–84. 10.1016/j.ejvs.2017.11.021 29306626

